# Supplementary material for: Improved yellow-green split fluorescent proteins for protein labeling and signal amplification
Source: PLoS One. 2020 Nov 23;15(11):e0242592. doi: 10.1371/journal.pone.0242592 (PMC7682878; doi:10.1371/journal.pone.0242592)
Supplement: S1 File — (PDF) [file pone.0242592.s001.pdf]

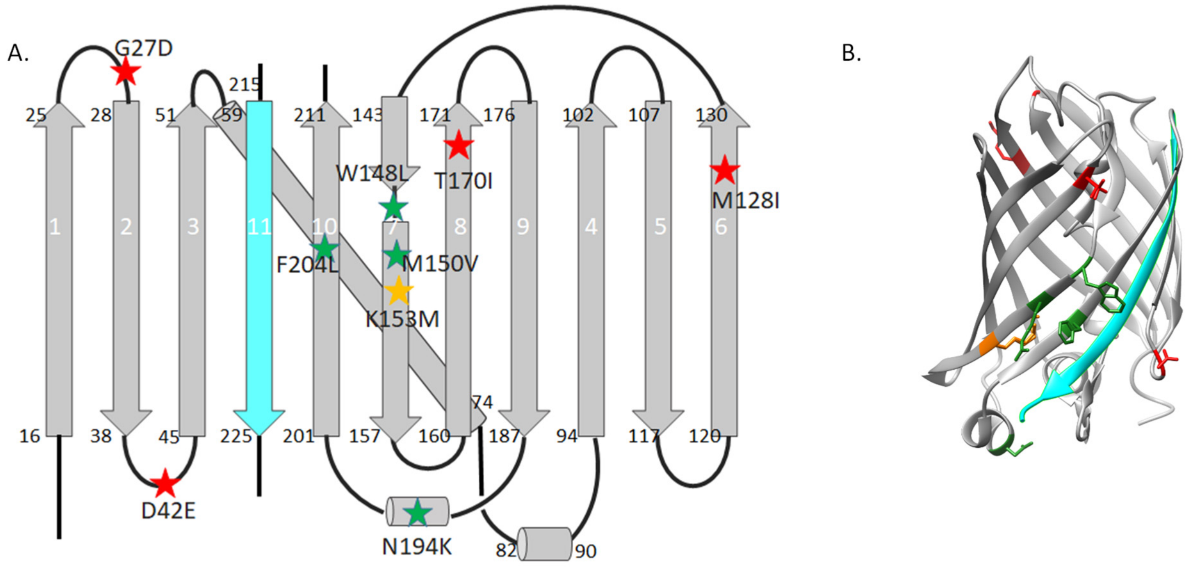

Fig. S1 mNG mutations in 2D and 3D structure. A. 2D structure of mNG. β-sheets are shown as arrows and α-helices are shown as cylinders. Mutations are shown as stars. B. structure of mNG (PBD entry 5LTR). A-B. mNG3A colored in green, mNG3K in red, and shared mutation in orange, β-strand 11 labelled in cyan.

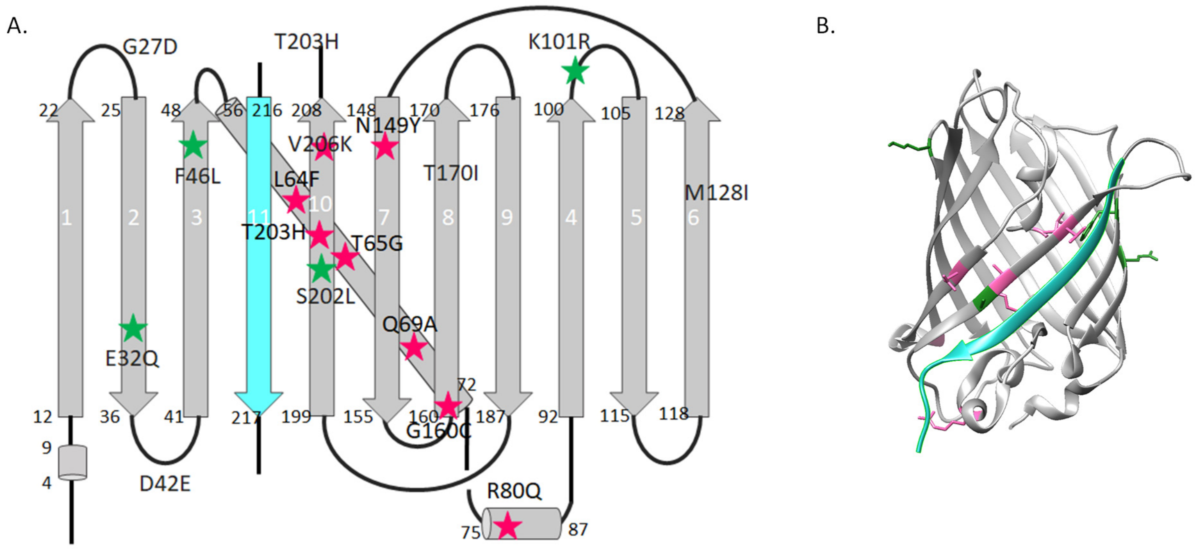

Fig S2. CloGFP mutations in 2D and 3D structure. A. 2D structure of sfGFP, β-sheets are shown as arrows and α-helices are shown as cylinders. Mutations are shown as stars. B, 3D structure of sfGFP from Protein Data Bank, ID 2B3P. A-B. mClover3 mutations in pink and CloGFP colored in green. β-strand 11 labelled in cyan.

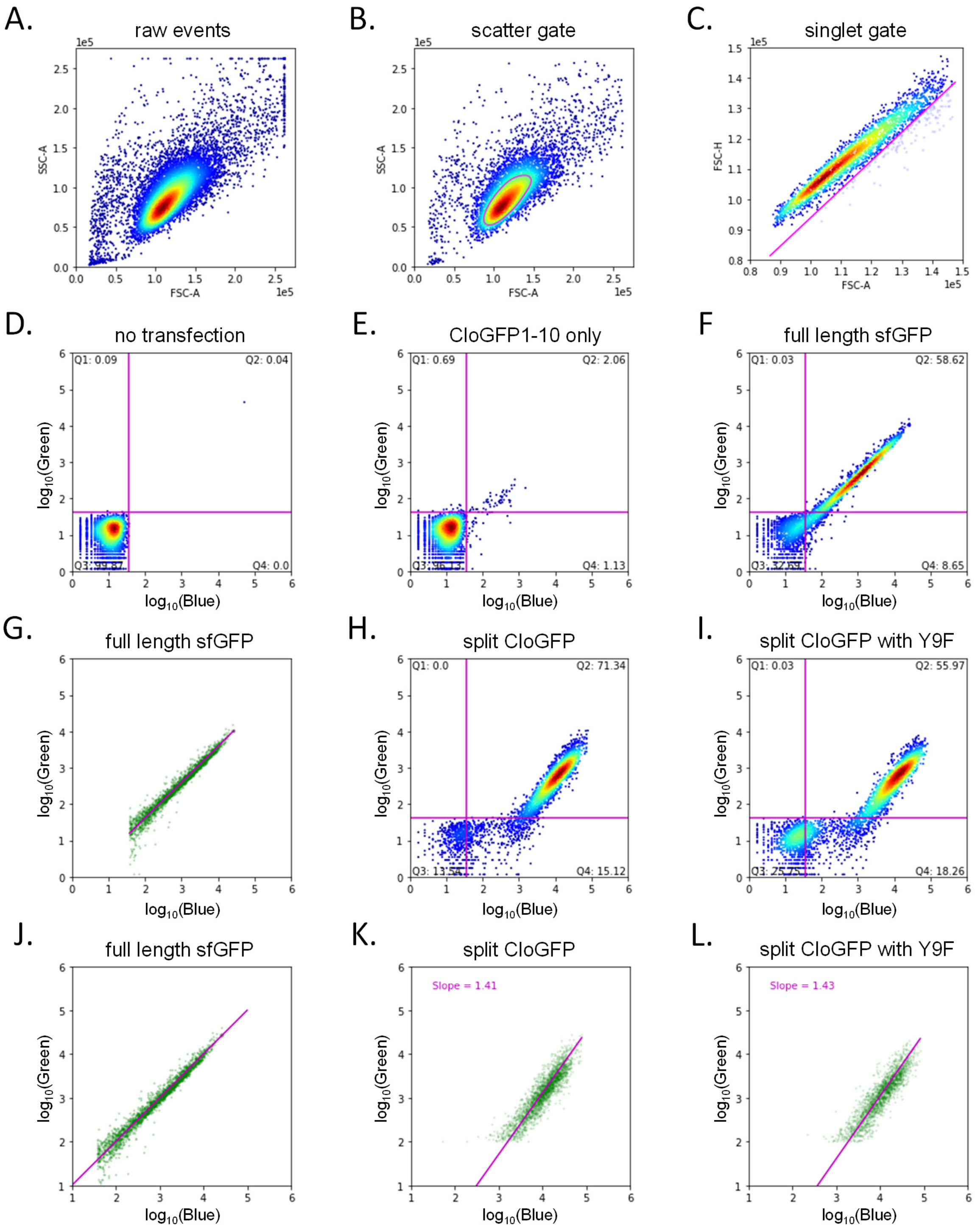

Fig S3. Flow Cytometry analysis of CloGFP$_{1-10}$ background in the 405 nm excitation (blue) and 488 nm excitation (green) channels. A. Raw flow cytometry events. B. Scatter gating. C. Singlet gating. D. Negative control without transfection. E.Transfected with CloGFP$_{1-10}$ only. F. Positive control with full length sfGFP. G. Fitting of full length GFP in BFP+ cells. H. CloGFP with wild type GFP$_{11}$. I. CloGFP with Y9F mutant GFP$_{11}$. J-L. Data from panels G-I rescaled for comparison.

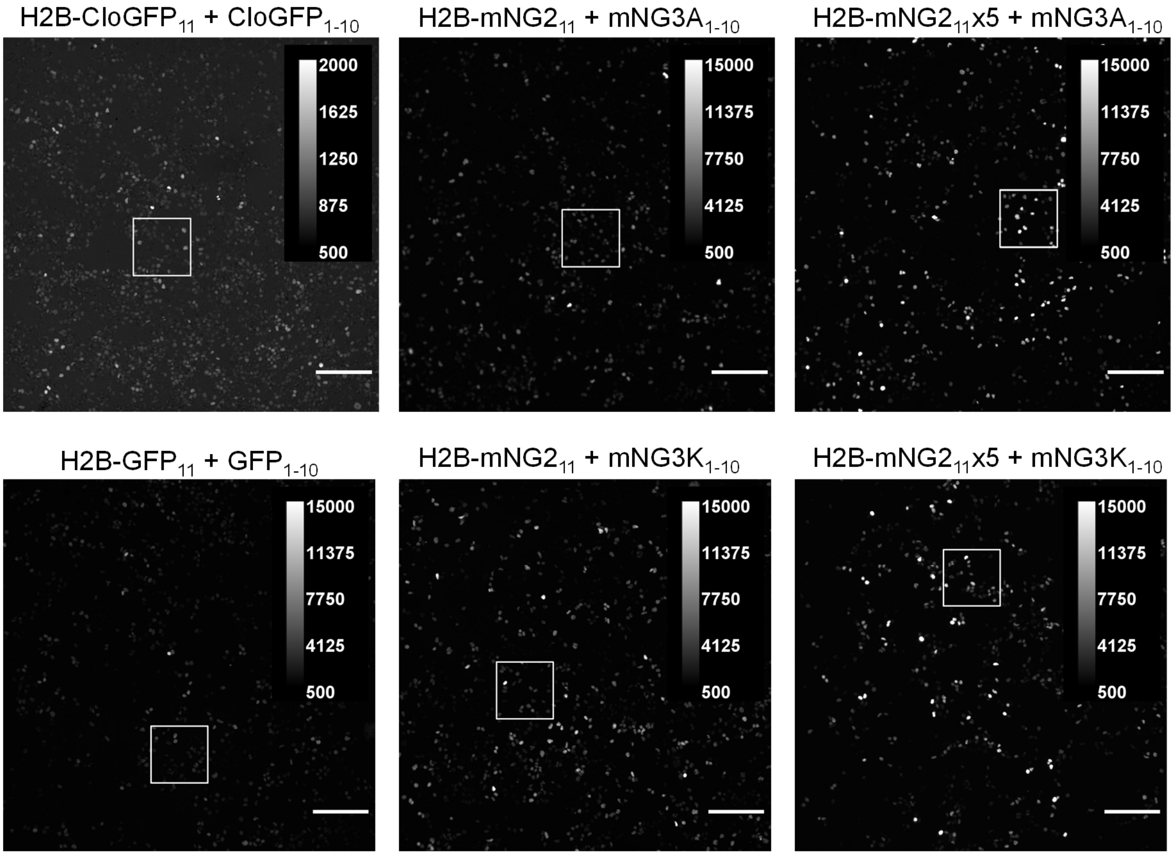

Fig S4. H2B labelling with new split proteins. Scale bars are 200 μm. Boxes indicate regions shown in Fig S5. Note that CloGFP is shown on a different intensity scale.

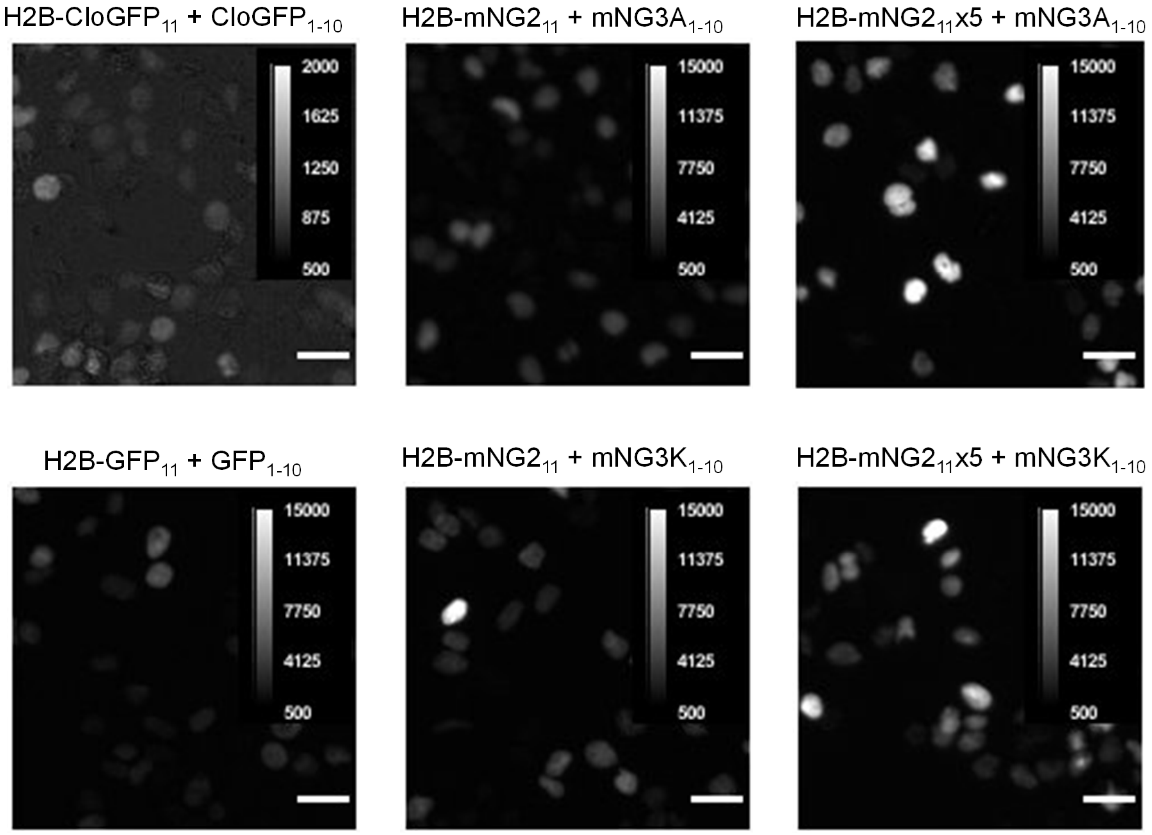

Fig S5. H2B labelling with new split proteins. Zoomed in images from the square box of Fig S4. White lines in the images are scale bars of 30 µm. Note the scale difference for the CloGFP compared with other images

Table S1. Mutation sites in each round of directed evolution for CloGFP.

Rounds	Genotype	Brightness
R0	CloGFP0	805
R1	K101R	3000
R2	K101R, S202L	15300
R3	G32Q, F46L, K101R, S202L<br>Y9F(GFP11)	34000
R4	G32Q, F46L, K101R, S202L<br>Y9F(GFP11)	30000/30700/35000/36000

Table S2 Protein sequences

Name	Sequences
mNG	MVSKGEEDNMASLPATHELHIFGSINGVDFDMVGQGTGNPNDGYEELNL KSTKGDLQFSPWILVPHIGYGFHQYLPYPDGMSPFQAAMVDGSGYQVHR TMQFEDGASLTVNYRYTYEGSHIKGEAQVKGTGFPADGPVMTNSLTAAD WCRSKKTYPNDKTIISTFKWSYTTGNGKRYRSTARTTYTFAKPMAANYLK NQPMYVFRKTELKHSKTELNFKEWQKAFTDVMGMDELYK
mNG2	MVSKGEEDNMASLPATHELHIFGSINGVDFDMVGQGTGNPNDGYEELNL KSTKGDLQFSPWILVPHIGYGFHQYLPYPDGMSPFQAAMVDGSGYQVHR TMQFEDGASLTVNYRYTYEGSHIKGEAQVMGTGFPADGPVMTNTLTAAD WCMSKKTYPNDKTIISTFKWSYTTVNGKRYRSTARTTYTFAKPMAANYLK NQPMYVFRKTELKHSMTELNFKEWQKAFTDMM
mNG3A	MVSKGEEDNMASLPATHELHIFGSINGVDFDMVGQGTGNPNDGYEELNL KSTKGDLQFSPWILVPHIGYGFHQYLPYPDGMSPFQAAMVDGSGYQVHR TMQFEDGASLTVNYRYTYEGSHIKGEAQVMGTGFPADGPVMTNTLTAAD LCVSKMTYPNDKTIISTFKWSYTTVNGKRYRSTARTTYTFAKPMAAKYLKN QPMYVLRKTELKHSMTELNFKEWQKAFTDMM
mNG3K	MVSKGEEDNMASLPATHELHIFGSINDVDFDMVGQGTGNPNEGYEELNL KSTKGDLQFSPWILVPHIGYGFHQYLPYPDGMSPFQAAMVDGSGYQVHR TMQFEDGASLTVNYRYTYEGSHIKGEAQVIGTGFPADGPVMTNTLTAAD WCMSKMTYPNDKTIISTFKWSYITVNGKRYRSTARTTYTFAKPMAANYLK NQPMYVFRKTELKHSMTELNFKEWQKAFTDMM
sfGFP	MSKGEELFTGVVPILVELDGDVNGHKFSVRGEGEGDATNGKLTLKFICTT GKLPVPWPTLVTTLTYGVQCFSRYPDHMKRHDFFKSAMPEGYVQERTIS FKDDGTYKTRAEVKFEGDTLVNRIELKGIDFKEDGNILGHKLEYNFNSHNV YITADKQKNGIKANFKIRHNVEDGSVQLADHYQQNTPIGDGPVLLPDNHYL STQSVLSKDPNEKRDHMVLLEFVTAAGITHGMDELYK
mClover3	MVSKGEELFTGVVPILVELDGDVNGHKFSVRGEGEGDATNGKLTLKFICT TGKLPVPWPTLVTTFGYGVACFSRYPDHMKQHDFFKSAMPEGYVQERTI SFKDDGTYKTRAEVKFEGDTLVNRIELKGIDFKEDGNILGHKLEYNFNSHY VYITADKQKNCIKANFKIRHNVEDGSVQLADHYQQNTPIGDGPVLLPDNHY LSHQSKLSKDPNEKRDHMVLLEFVTAAGITHGMDELYK
CloGFP0.2	MSKGEELFTGVVPILVELDGDVNGHKFSVRGEGEGDATIGKLTLKFICTTG KLPVPWPTLVTTFGYGVACFSRYPDHMKQHDFFKSAMPEGYVQERTISF KDDGKYKTRAVVKFEGDTLVNRIELKGTDFKEDGNILGHKLEYNFNSHYV YITADKQKNCIKANFTVRHNVEDGSVQLADHYQQNTPIGDGPVLLPDNHY LSHQTKLSKDPNEKRDHMVLHEYVNAAGIT
CloGFP	MSKGEELFTGVVPILVELDGDVNGHKFSVRGQGEGDATIGKLTLKLICTTG KLPVPWPTLVTTFGYGVACFSRYPDHMKQHDFFKSAMPEGYVQERTISF RDDGKYKTRAVVKFEGDTLVNRIELKGTDFKEDGNILGHKLEYNFNSHYV YITADKQKNCIKANFTVRHNVEDGSVQLADHYQQNTPIGDGPVLLPDNHY LLHQTKLSKDPNEKRDHMVLHEYVNAAGIT
